# Emissions of DEHP‐free PVC flooring

**DOI:** 10.1111/ina.12591

**Published:** 2019-09-11

**Authors:** Emmanuelle Castagnoli, Peter Backlund, Oskari Talvitie, Tapani Tuomi, Arja Valtanen, Raimo Mikkola, Hanna Hovi, Katri Leino, Jarek Kurnitski, Heidi Salonen

**Affiliations:** ^1^ Department of Civil Engineering Aalto University Espoo Finland; ^2^ Finnish Institute of Occupational Health Helsinki Finland; ^3^ RTC Vahanen Turku Oy Turku Finland; ^4^ The Building Information Foundation RTS sr Helsinki Finland; ^5^ Tallinn University of Technology Tallinn Estonia

**Keywords:** 2‐ethylhexanol, C9‐alcohol, flooring, phthalates, PVC, TVOC

## Abstract

Degrading 2‐ethylhexyl‐containing PVC floorings (eg DEHP‐PVC floorings) and adhesives emit 2‐ethylhexanol (2‐EH) in the indoor air. The danger of flooring degradation comes from exposing occupants to harmful phthalates plasticisers (eg DEHP), but not from 2‐EH as such. Since the EU banned the use of phthalates in sensitive applications, the market is shifting to use DEHP‐free and alternative types of plasticisers in PVC products. However, data on emissions from DEHP‐free PVC floorings are scarce. This study aimed at assessing the surface and bulk emissions of two DEHP‐free PVC floorings over three years. The floorings were glued on the screed layer of concrete casts at 75%, 85%, and 95% RH. The volatile organic compounds (VOCs) were actively sampled using FLEC (surface emissions) and micro‐chamber/thermal extractor (µ‐CTE, bulk emissions) onto Tenax TA adsorbents and analyzed with TD‐GC‐MS. 2‐EH, C9‐alcohols, and total volatile organic compound (TVOC) emissions are reported. Emissions at 75% and 85% RH were similar. As expected, the highest emissions occurred at 95% RH. 2‐EH emissions originated from the adhesive. Because the two DEHP‐free floorings tested emitted C9‐alcohols at all tested RH, it makes the detection of flooring degradation harder, particularly if the adhesive used does not emit 2‐EH.

**Practical implications**

- DEHP‐free floorings emit mixtures of long‐chain alcohols instead of 2‐EH which is emitted from DEHP‐PVC floorings.
- The studied DEHP‐free floorings naturally emitted C9‐alcohols at all tested RH (RH ≥ 75%).
- Assessing the degradation of PVC floorings in building is more difficult when they no longer emit 2‐ethylhexanol in the indoor air.

## 1 INTRODUCTION

Polyvinyl chloride (PVC) floorings are common in residential and commercial buildings.^1^, ^2^ New plastic floorings can significantly pollute indoor air by emitting manufacturing residues—as volatile organic compounds (VOCs).^3^ However, this type of pollution is only temporary and can be dealt with by increasing the ventilation rate. The degradation of plastic floorings is much more concerning. Excess water in a concrete slab can trigger degradation of the plastic flooring—for example, when flooring is laid on wet concrete, or in the case of (hidden) moisture damage. Hydrolysis of the plastic flooring will initiate the migration of its plasticiser (=additive) from the polymer matrix to, for example, the indoor air and dust.^3^, ^4^ Exposure to plasticiser can endanger the health of building occupants.

A finished plastic product may contain up to 40% plasticisers.^5^, ^6^ In the past, most PVC floorings were manufactured using DEHP plasticiser.^7^, ^8^ Phthalate plasticisers, such as DEHP, DIDP, and DINP, are dangerous, endocrine‐disrupting chemicals, suspected carcinogens, and causative agents of airway diseases and asthma in children. They are capable of leaching into blood bags,^9^, ^10^, ^11^, ^12^ food packaging,^12^, ^13^, ^14^ and indoor dust.^4^, ^8^, ^12^, ^15^, ^16^, ^17^, ^18^, ^19^, ^20^, ^21^, ^22^, ^23^ In the EU, phthalate plasticisers can no longer be used for sensitive applications.^22^, ^24^ Therefore, new, non‐toxic, DEHP‐free plasticisers such as DINCH are emerging on the market.^22^, ^25^, ^26^

Plasticisers constitute different hydrocarbon chains: 2‐ethylhexyl (for DEHP and DEHA plasticisers), C8‐C10 alkyl isomers (for DINCH and DINP plasticisers), C9‐C11 alkyl isomers (for DIDP plasticiser), or alkyl groups (for triglycerides plasticisers). The shorter and non‐polar the hydrocarbon chain is, the more easily it leaches from the PVC product.^5^ Under hydrolysis conditions, 2‐ethylhexyl will degrade into 2‐ethylhexanol (2‐EH), the long alkyl isomers will degrade into various long‐chain alcohols (eg C9‐alcohols), and the alkyl groups will degrade into carboxylic acids. Nowadays, the dangerous DEHP is replaced by non‐phthalates (DINCH, DEHA, and triglycerides) and longer hydrocarbon chain phthalate plasticisers (DIDP and DINP).

Degradation of DEHP‐PVC floorings has been extensively studied. In addition to the relative humidity (RH), other parameters affect flooring emissions. For instance, a rise of pH (alkaline pH, pH > 7) in the concrete slab increases the hydrolysis rate.^27^ However, adding a screed layer between the concrete slab and glue/flooring interface will act as a buffer and prevent this rise of pH.^27^, ^28^ Temperature also affects flooring emissions, for example, when room temperature increases or when there is a presence of an in‐floor heating system.^16^, ^29^, ^30^

To prevent premature degradation of plastic floorings, the Finnish protocol RT 14‐10984 recommends laying new plastic floorings when the concrete slab surface is <75% RH and bulk is <85% RH.^31^ Moreover, selecting low‐emitting building material will limit the impact on the indoor air quality. The voluntary Finnish M1 label is a classification intended to help customer discriminate between the building material emissions.^32^ It classifies building material from lowest (M1) to highest (M3) emitting. The emissions are tested according to standards ISO 16000‐9:2006 and EN 16516:2017 after a 28‐day aging period in a ventilated chamber at 50 ± 5% RH and 23 ± 1°C.^33^, ^34^

There are various sources of 2‐EH emissions: microbes, ethylhexyl acrylate‐containing adhesives, and degrading PVC floorings—for example, containing 2‐ethylhexyl plasticisers, or trimellitate, adipate and terephthalate esters plasticisers.^35^, ^36^, ^37^ Exposure to indoor 2‐EH is not a problem unless the concentration exceeds the irritation potency of 175 µg/m^3^ (expressed as RD50 values), which provokes irritation of the respiratory tract and asthma symptoms.^35^, ^38^ However, the detection of 2‐EH is a strong indicator of DEHP‐PVC flooring degradation and indirect proof of occupant exposure to dangerous DEHP as well as to possible microbial growth.^39^, ^40^

Alternative plasticisers do not degrade into one major chemical like DEHP does, but produce a mixture of long‐chain alcohols. Since the shift to alternative plasticisers, the health risks arising from moisture damage, and possibly microbial growth, are harder to evaluate. Data on degradation of alternative plasticisers are scarce.^41^ Because the nature of the replacing plasticisers (and their hydrocarbon chains) is different, it is necessary to study the degradation of such PVC floorings (as emissions). Is it possible that under the same conditions, DEHP‐free PVC hydrolyzes more readily than PVC containing DEHP? Are we sure that DINCH and other aliphatic esters are suitable new additives in floorings? How should we assess floor damage when the compound that indicates degradation is no longer present in the flooring?

In this study, the emissions of two DEHP‐free PVC floorings laid at 75%, 85%, and 95% RH were investigated over three years. Emissions were monitored as 2‐ethylhexanol, C9‐alcohols, and total volatile organic compounds (TVOC), actively sampled on Tenax TA and analyses with gas chromatography‐mass spectrometry. The surface and bulk emissions were simultaneously monitored. The field and laboratory emission cell (FLEC) sampling method was selected to monitor surface emissions as it is the method used to investigate building material emissions by indoor air inspectors and determines how much is emitted from the building material into the indoor air. The bulk emissions from the plastic floorings were assessed using a micro‐chamber/thermal extractor device (µ‐CTE) and represent both surface emissions and emissions stemming from the underlying layers of the PVC floorings.

## 2 MATERIAL AND METHODS

The experimental work was conducted in the laboratory of the Finnish Institute of Occupational Health (FIOH), Finland. Laboratory Services is a testing laboratory T013 accredited by FINAS Finnish Accreditation Service, accreditation requirement ISO/IEC 17025 and the VOC analysis covered by this accreditation.

### 2.1 PVC‐coated concrete slabs

**Figure 1:**
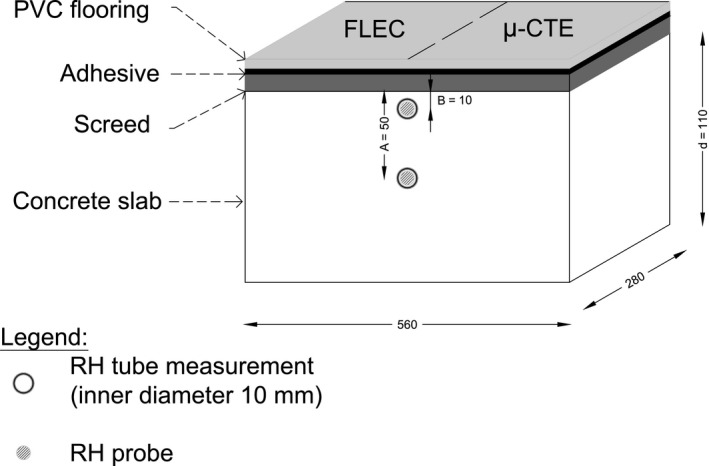
Details of one coated concrete slab with the location of the field and laboratory emission cell (FLEC), micro‐chamber/thermal extractor (µ‐CTE), and relative humidity (RH) measurements (not on scale, dimensions reported in mm)

Six concrete slabs were cast into anodized aluminum molds of dimension 280 × 560 × 110 mm. Tubes of 10 mm inner diameter were inserted at 10 mm (surface tube) and 50 mm (bulk tube) below the surface of each cement slab (Figure 1). Cement‐based screed was spread over the surface of the concrete slabs. Two types of PVC floorings were adhered onto the screed with water‐based adhesive (approximately 207 g/m^2^) when the concrete slabs reached 75%, 85%, or 95% RH (measured from the surface tube). The slabs were stored for three years in a clean laboratory under normal room conditions (temperature‐controlled room, 21 ± 3°C, no RH data available). Only the lowest emitting building material (M1 classified) was selected in this study, and further specifications are listed in Table 1.

**Table 1:** Material specification

Material	Emission class^a^	Description
PVC 1	M1	Sound insulating vinyl flooring. Thickness 2.6 mm and abrasion surface thickness of 0.25 mm
PVC 2	M1	Wear‐resistant vinyl flooring. Reinforced surface of 2.4 mm thickness and abrasion surface thickness of 0.40 mm
Adhesive	M1	Water‐based adhesive acryl dispersion (for example to fix vinyl flooring)
Screed	M1	Cement based, finishing compound for concrete floors. Easy spreading and fast curing. Suitable for layer thicknesses of 1‐10 mm. Special cement mixture with sand and limestone aggregates (<0.3 mm), low pH (10.5‐11)
Concrete		Portland cement with sand aggregates of size 0‐4 mm

^a^ Emission class specified from the supplier.

### 2.2 RH measurements

**Figure 2:**
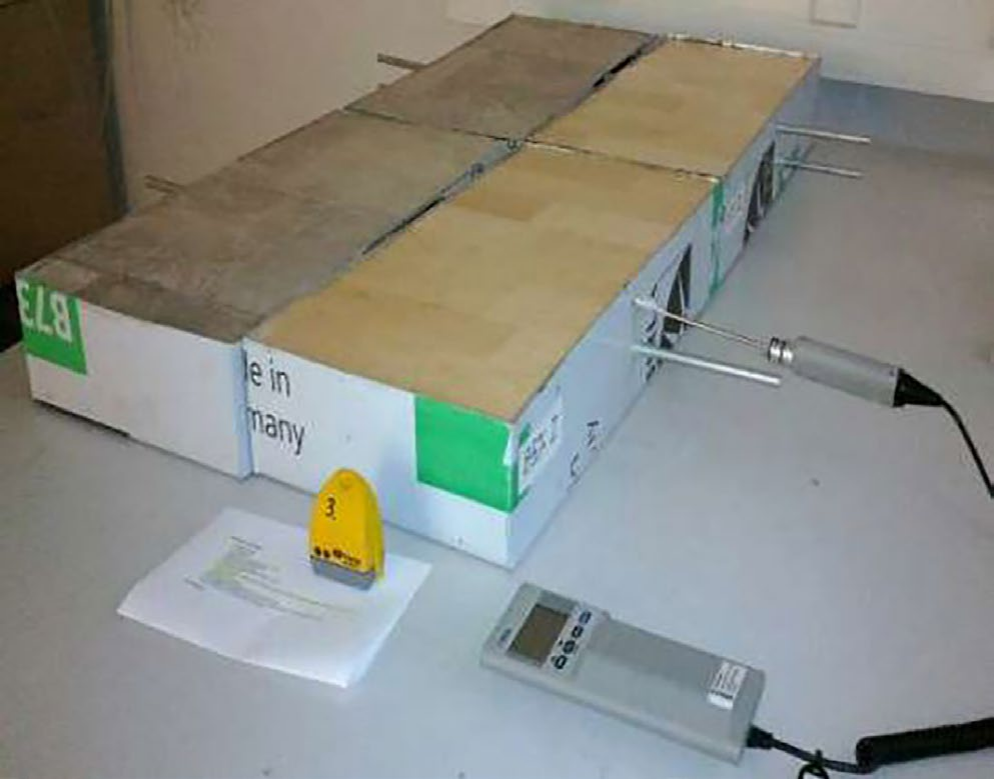
Measurement of the surface RH of one of the coated concrete slab

Vaisala HMI41 humidity and temperature probe (serial number C4910014, Helsinki, Finland) was used to measure the RH. The Vaisala probe was sealed overnight in the tube (with Parafilm M) before reading its value. Between measurements, round bars of 10 mm diameter were inserted into the tubes to prevent moisture leakage. Figure 2 illustrates surface RH measurement.

In Finland, the national protocol RT 14‐10984 provides guidelines for measuring the RH of a concrete structure.^31^ Generally, the RH is assessed from two depths (A and B) determined by the structure thickness (d) and the drying conditions of the structure.^31^ In case of a bi‐directional drying, the depth measurement B (or “surface” measurement) is B = 0.4 × A and the depth measurement A (or “bulk” measurement) is 20% of the structure thickness (A = 0.2 × d). When the drying takes place in one direction only, the measuring depth B remains unchanged but the measurement depth A is 40% of the structure thickness for a maximal value of 70 mm (A = 0.4 × d, with A_max_ < 70 mm). With PVC floorings, the protocol recommends to glue the flooring when the RH is A < 85% RH and B < 75% RH (with B = 0.4 × A).^31^

### 2.3 Measurement of surface and bulk emissions

The VOC (including 2‐EH, C9‐alcohols, and TVOC) emissions were monitored with FLEC (surface emissions) and µ‐CTE (bulk emissions) 1‐36 months after laying of the PVC floorings. The same side of the concrete slab was used to monitor the surface emissions, after wiping down dust (FLEC, Figure 1). The opposite side of the slab was reserved to cut pieces of PVC flooring (4 × 4 cm) to measure the bulk emissions (µ‐CTE, Figure 1). To avoid loss of moisture, the missing pieces of floorings were replaced with pieces of aluminum. The surface and bulk emissions were simultaneously monitored.

The emission samples were collected following the FIOH guidelines. FLEC sampling is based on ISO 16000‐10:2011.^42^ The bulk sampling is based on an in‐house method, in which the µ‐CTE was set to 25°C. The emission samples were actively sampled on Tenax TA adsorbent tubes and analyzed at FIOH following ISO 16000‐6:2011 using thermal desorber‐gas chromatography‐mass spectrometry (TD‐GC‐MS).^43^ The accuracy, repeatability, and method uncertainties are compound‐dependent. The uncertainty of the VOC measurements method is, on average, 30%. The accuracy of, for instance, 2‐EH and 1‐butanol is 100 and 88%, respectively, and the repeatability 10% (2‐EH) and 7% (1‐butanol). The concentrations are determined using pure calibrators in 5‐8 concentration points. On average the limits of quantitation are of 4 ng per sample. Blanks and control samples are run throughout the analysis sequences, the results of which are subjected to strict margins. In practice, as pertains the background concentration of, for instance, 2‐EH and C9‐alcohols, they are below the limits of quantitation. FIOH has for years participated in quality assurance rounds (proficiency testing) organized by both IFA (Institut für Arbeitsschutz der Deutschen Gesetzlichen Unfallversicherung, Germany) and LGC (LGC Standards Gmbh, Germany) with excellent results. In addition, FIOH organizes a yearly proficiency testing scheme in co‐operation with the Finnish Environment Institute, in which FIOH is the reference laboratory.

The portable FLEC chamber is the standard method used by indoor air inspectors to investigate on‐site building material surface emissions.^42^ It represents the VOC concentrations per unit time and surface area are in the prevailing conditions, as pertaining to compounds emitting from or—to a lesser extent—through the surface material. µ‐CTE is a fast and cost‐effective method to control the VOC emissions of products.^44^ The bulk emissions pertain to both surface emissions and emissions stemming from the underlying layers. Therefore, in real‐life situations emissions stem from both the surface of the floor coverings as well as from underneath the floor coverings via the sides and other points, where the covering discontinues for some reason or another. Emissions originating from, for instance, moisture‐dependent decomposition of the glue or bottom surface of the floor coverings emit poorly through the front side of the coverings. These may, however as mentioned above, find other emission routes. Hence, because gaseous emissions always take “the easiest route of travel,” we wanted to measure simultaneously both bulk and surface emissions.

## 3 RESULTS

### 3.1 RH

**Figure 3:**
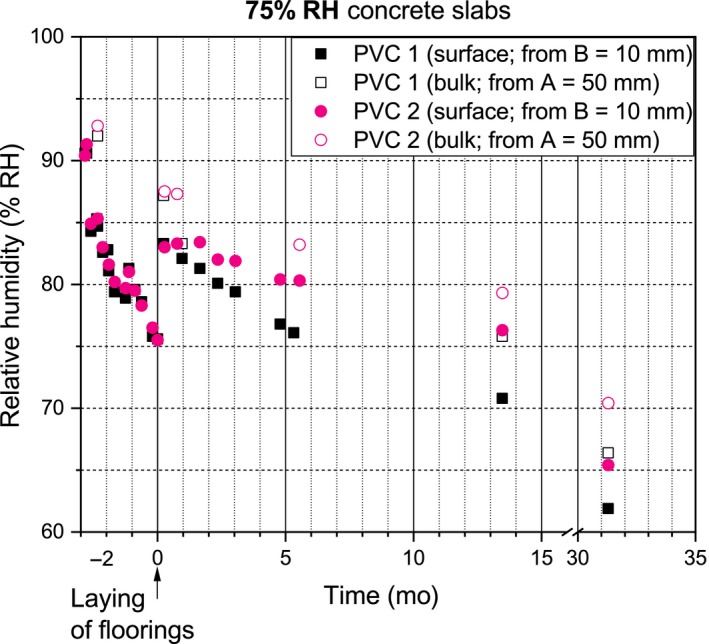
RH values of the 75% RH concrete slabs

**Figure 4:**
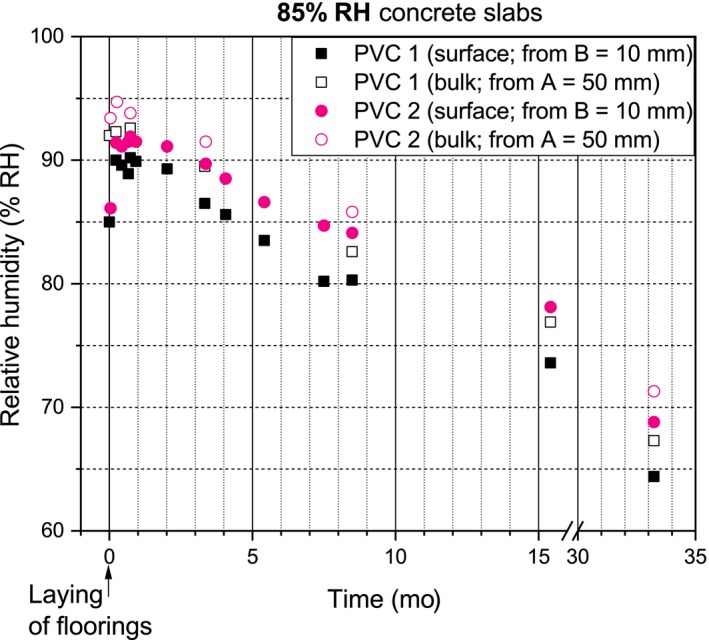
RH values of the 85% RH concrete slabs

**Figure 5:**
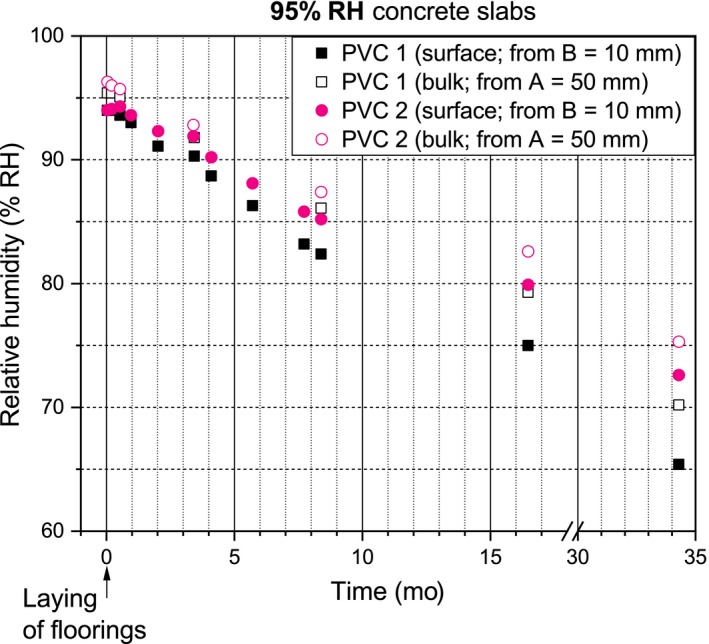
RH values of the 95% RH concrete slabs

The RH profiles of the concrete slabs are presented in Figures 3, 4, and 5. Laying of the PVC floorings initiated moisture redistribution in the 75% and 85% RH concrete slabs which increased their surface and bulk RH. After the laying and moisture redistribution, the surface and bulk RH of the 75%, 85%, and 95% RH‐covered concrete slabs continuously decreased. The PVC 2‐covered concrete slabs consistently had higher surface and bulk RH than the PVC 1‐covered concrete slabs.

### 3.2 Emissions from the PVC floorings

**Figure 6:**
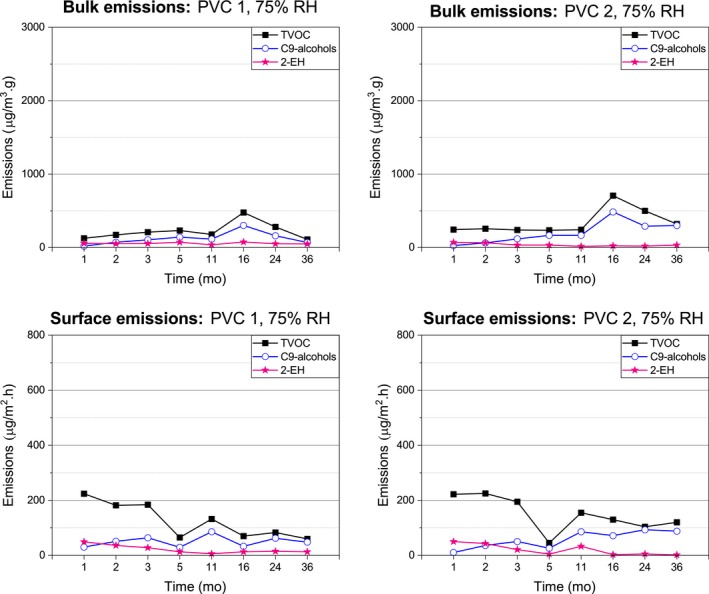
Emissions of the 75% RH‐covered concrete slabs

**Figure 7:**
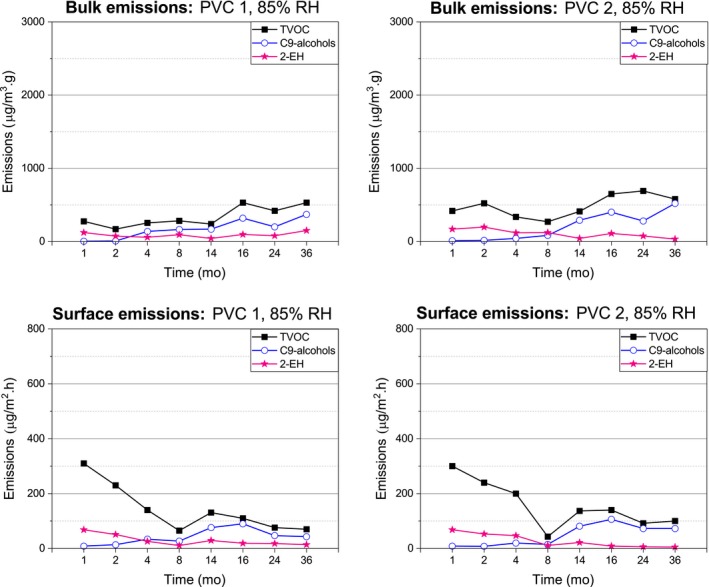
Emissions of the 85% RH‐covered concrete slabs

**Figure 8:**
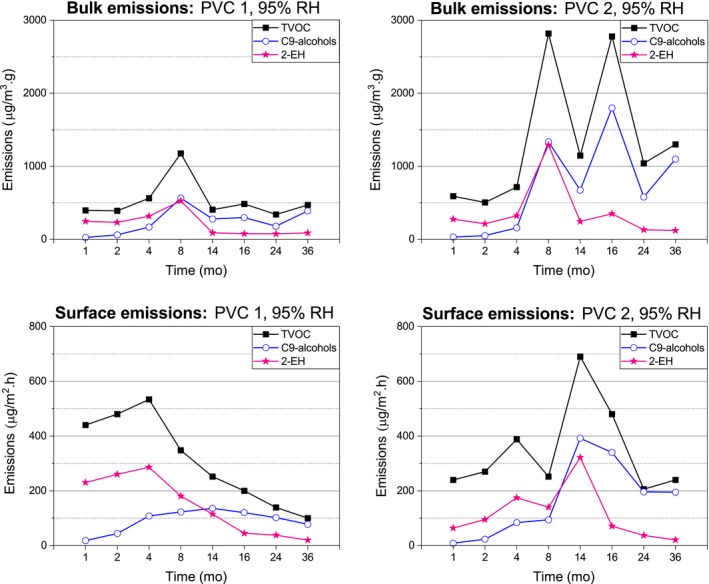
Emissions of the 95% RH‐covered concrete slabs

The Figures 6, 7, and 8 represent the emissions of C9‐alcohols, 2‐EH, and TVOC from the covered concrete slabs. The concentrations are reported as toluene equivalents.

#### 3.2.1 C9‐alcohol emissions

Similar concentration of C9‐alcohols was emitted by the 75 and 85% RH‐covered concrete slabs, with constantly slightly higher emissions for the PVC 2‐covered than the PVC 1‐covered concrete slabs. The surface emissions of the 75% and 85% RH‐covered concrete slabs slowly increased during the first 11 and 16 months, respectively, and then, they decreased for the PVC 1‐covered but remained constant for the PVC 2‐covered concrete slabs. The PVC‐covered 75% and 85% RH concrete slabs emitted similar concentration of bulk C9‐alcohols during the first 16 months, then, the 75% RH‐covered concrete slabs emissions decreased and those of the 85% RH‐covered concrete slab increased (especially noticeable at month 36).

The 95% RH‐covered concrete slabs exhibited the highest C9‐alcohol emissions. The surface emissions of the PVC 1‐covered concrete slab progressively increased during the first 14 months and from then slowly decreased. The PVC 2‐covered concrete slab gradually emitted increasing surface C9‐alcohols during the first eight months before a drastic augmentation at month 14 (over twice‐higher emissions than with the PVC 1‐covered concrete slab) followed by a slow decrease of emissions. The PVC 1‐ and PVC 2‐covered concrete slabs emitted similar concentration of bulk C9‐alcohols during the first four months, after which the bulk emissions periodically increased/decreased. After the fourth month, the bulk emissions were much more significant in the case of the PVC 2‐covered concrete slab.

#### 3.2.2 2‐EH emissions

The 75 and 85% RH‐covered concrete slabs emitted the highest 2‐EH concentrations at the beginning of the study, these emissions were similar and generally decreased over time. At month 36, the 85% RH PVC 1‐covered concrete slabs showed an increase of the 2‐EH bulk emissions.

The highest 2‐EH emissions were detected from the 95% RH‐covered concrete slabs. The surface emission profiles between the PVC 1‐ and PVC 2‐covered concrete slabs were different. The 95% RH PVC 1‐covered concrete slab emitted high concentrations of surface 2‐EH right from the beginning of the study. These emissions kept increasing very slowly during the first four months. From month four, the surface 2‐EH emissions of the PVC 1‐covered concrete slab gradually decreased. On the other hand, the PVC 2‐covered concrete slab progressively emitted surface 2‐EH during the first 14 months (as high as for the PVC 1‐covered concrete slab after four months) and these emissions sharply decreased between months 14 and 16, and from month 16 slowly decreased until the rest of the study.

The bulk emission profiles of the 95% RH‐covered concrete slabs were similar. The bulk 2‐EH emissions gradually increased during the first eight months with both PVC floorings, but the increase of bulk emissions for the PVC 2‐covered concrete slab between months 4 and 8 was very sharp like its decrease between months 8 and 14 with both PVC‐covered concrete slabs. The bulk 2‐EH emissions after month 14 were generally constant with both PVC‐covered concrete slabs.

#### 3.2.3 TVOC emissions

The TVOC emission profiles of the 75% and 85% RH‐covered concrete slabs were alike, although the bulk TVOC emissions were slightly higher with the PVC 2‐covered concrete slab. During the first few months of the study, over half of the initial TVOC surface emissions detected (during the first five months in the case of the 75% RH‐covered concrete slab and the first eight months in the case of the 85% RH‐covered concrete slab) originated from other VOCs than the 2‐EH and C9‐alcohols. Over time, the combination of 2‐EH and C9‐alcohol emissions detected from the 75% and 85% RH‐covered concrete slabs accounted for most of the TVOC surface emissions, particularly the C9‐alcohols. This trend was also true for the TVOC bulk emissions.

The TVOC emission profiles of the 95% RH‐covered concrete slabs were different from the 75% and 85% RH‐covered concrete slabs. The PVC 1‐covered concrete slab emitted more surface TVOC (attributable to the higher 2‐EH emissions) during the first four months than the PVC 2‐covered concrete slab. Overall, initial TVOC emissions were attributed to the 2‐EH emissions until month eight (except in the case of the PVC 1‐covered slab surface emissions, which was four months only). Then, the C9‐alcohols became the dominant contributor of the TVOC emissions. From month 8, the PVC 2‐covered slab showed high TVOC emissions, especially from the bulk sample.

PVC 1 mainly emitted 40% of nonanal, 27% of hexanal, and 6% of 2‐EH (Supplementary material figure S1, S2 and S3). PVC 2 emitted 17% of 1,2 propanediol, 9% of nonanal, 7% of hexanal, 3% of 2‐EH, and other compounds (Supplementary material figure S2). The adhesive mainly emitted ethylhexyl acrylate (Supplementary material figure S3).

## 4 DISCUSSION

C9‐alcohols were emitted by each of the covered concrete slabs. This indicates that the PVC floorings contained the C9‐alkyl type of plasticisers (Supplementary material figures S1 and S2). These emissions were expected for the PVC floorings laid at high initial RH (eg >85% RH) since degradation is more likely to occur. The 75% RH slabs also emitted C9‐alcohols. Therefore, it was concluded that the two investigated DEHP‐free PVC floorings emitted C9‐alcohols even at 75% RH. For each of the initial laying RH ranges (meaning: 75%, 85%, and 95% RH), the C9‐alcohol emissions increased and fluctuated during the three‐year study, although the RH of the concrete slabs constantly decreased. Considering the preliminary results presented in this study, C9‐alcohol emissions alone might not be a suitable indicator of moisture damage—for example, in the case of PVC 1 FLEC emissions, the C9‐alcohol emission level was just below 100 μg/m^2^.h at 75% and 85% RH, while at 95% RH it was just above that value. It would be hard to conclude whether hidden moisture damage is occurring in the concrete based on C9‐alcohol emissions alone.

The 2‐EH emissions were attributed to the water‐based adhesive acryl dispersion (Supplementary material figure S3).^45^, ^46^, ^47^, ^48^ The emissions from residual 2‐ethylhexyl present in the adhesive were observed with the 75% and 85% RH, and the 95% RH PVC 1‐covered concrete slabs.^45^, ^46^, ^47^, ^48^ Approximately six months after laying, the 2‐EH emissions increased due to hydrolysis of the water‐based adhesive for the 85% RH PVC 1 (at month 36), and the 95% RH‐covered concrete slabs. Only one type of adhesive was considered here. The 2‐EH emissions were attributed to primary emissions and hydrolysis products at critical RH (95% RH) of the water‐based adhesive acryl dispersion.

The TVOC emissions were used to estimate to which level the 2‐EH and C9‐alcohols contributed to the total VOC emissions. Initially, the 75% and 85% RH‐covered concrete slabs emitted VOCs other than 2‐EH and C9‐alcohols, present as impurities in the PVC flooring or the adhesive. On the contrary, the 95% RH‐covered concrete slabs initially emitted 2‐EH. In the long term, each of the covered concrete slabs predominantly emitted C9‐alcohols.

The emissions obtained by µ‐CTE analysis were run at ambient temperature to avoid variation due to possible sample heterogeneity.^44^, ^49^, ^50^, ^51^ The variations between FLEC and µ‐CTE emissions and PVC 1 and PVC 2 emissions might result from different composition and sensitivity to degradation of the PVC floorings, as well as non‐uniform moisture content within the slabs. For example, the thicker, wear‐resistant flooring structure of PVC 2 impeded the diffusion of 2‐EH from the adhesive into the indoor air.^52^, ^53^ Moreover, it hampered the drying process by trapping moisture for longer than PVC 1 does, made it more sensitive to hydrolysis, and produced higher C9‐alcohol and TVOC emissions. Therefore, the higher RH of the PVC 2‐covered concrete slabs might be the reason for the extreme variation within bulk samples.

In Finland, the RT 14‐10984 protocol recommends floorings be laid when the concrete surface RH, B, is <75% RH (B protocol = 18 mm ≠ B study = 10 mm) and bulk RH, A, is <85% RH (A protocol = 44 mm ≈ A study = 50 mm; RH tube inner diameter = 10 mm). The B study was closer to the concrete surface than recommended. In our study, the values were as follows: 75% RH slabs (B = 75% RH/ A = 87% RH), 85% RH slabs (B = 85% RH/A = 93% RH), and 95% RH slabs (B = 95% RH/A = 96% RH). The 75% RH slabs were close to protocol, whereas the 85% RH and 95% RH slabs were very wet slabs. Emissions from the 75% and 85% RH slabs were quite low compared to their RH. High emissions at critical RH (95% RH) were expected.

The presence of 2‐EH in the indoor air is used to track the degradation of phthalate‐containing PVC floorings. The Finnish Ministry of Social Affairs and Health has set the action limit to 10 µg/m^3^ (≈13 μg/m^2^.h, calculated with the M1 protocol).^54^ This is the concentration at which necessary actions should be taken to eliminate or limit the harm it may cause.^54^ In this study, the action limit was exceeded during the first 5 months with the 75% RH concrete slabs, the first eight months for the 85% RH PVC 2‐covered slab, and throughout the study period for the 85% RH PVC 1‐covered concrete slab (except for month 8), and the 95% RH‐covered concrete slabs. The irritation potency of 2‐EH (expressed as RD50 values) is 175 µg/m^3^ (≈219 μg/m^2^.h, calculated with the M1 protocol).^55^ It was exceeded with the 95% RH‐covered concrete slabs, during the first 8 months for the PVC 1‐covered slab and only at month 14 for the PVC 2‐covered concrete slab. The FIOH sets the limit for TVOC emissions in offices to 250 μg/m^3^ (≈312 μg/m^2^.h, calculated with the M1 protocol).^55^ This value exceeded the first month only for the 85% RH‐covered concrete slabs, and during the first 14 and 24 months for the PVC 1‐ and PVC 2‐covered 95% RH concrete slabs, respectively. The studied PVC floorings also emitted aldehydes. PVC 1 emitted 27% of hexanal and 40% of nonanal, and PVC 2 emitted 9% of hexanal and 7% of nonanal (Supplementary material figures S1 and S2). Aldehydes are odor and sensory irritation agents—for example, hexanal.^56^ Campioli et al (2017) had reported cytotoxicity of DINCH plasticiser; however, DINCH does not migrate from PVC as easily as the toxic DEHP does.^5^, ^57^ There are no data on the inhalation toxicity of C9‐alcohols, but they have been reported as low toxicity compounds.^58^

In conclusion, this preliminary study shows that C9‐alcohols were emitted above 75% RH. Even though the study was limited to only two DEHP‐free PVC floorings, it indicates that C9‐alcohol emissions seem to be a property of the DEHP‐free PVC floorings investigated. This makes the detection of flooring degradation, and of hidden floor moisture damage, more difficult, particularly if the adhesive used does not contain/emit 2‐EH. When using 2‐ethylhexyl‐based floorings (meaning DEHP) or adhesives, the detection of flooring degradation and of hidden floor moisture damage is straightforward and possible with monitoring one single compound, 2‐EH, in the indoor air. On the contrary, the new long‐chain alcohol‐based flooring materials (here, C9‐alcohol‐based floorings) do not degrade into one single 2‐EH compounds anymore but emit a mixture of long‐chain alcohols. Therefore, if the new flooring materials do not emit 2‐EH, and if at the same time the adhesive used do not either emit 2‐EH, it will not be anymore possible to use 2‐EH emissions as an indicator of degradation or of hidden moisture damage. The new ester additives used to replace the phthalate plasticiser undergo similar degradation than DEHP, but the analysis and identification of their degradation products are much more difficult than with 2‐EH (eg, originating from the hydrolysis of DEHP‐PVC). This study was limited to only two PVC floorings, and neither the effect of pH or temperature were studied here.

In future, it will be important to study the emissions of a higher number of PVC floorings, but also to determine the effects of the concrete pH level, of using a screed layer, or of the room temperature.

## CONFLICT OF INTEREST

The authors declare no conflict of interest. The funders had no role in the design of the study; in the collection, analyses, or interpretation of data; in the writing of the manuscript, or in the decision to publish the results.
